# Preoperative nomogram for microvascular invasion prediction based on clinical database in hepatocellular carcinoma

**DOI:** 10.1038/s41598-021-93528-7

**Published:** 2021-07-07

**Authors:** Shuqi Mao, Xi Yu, Yong Yang, Yuying Shan, Joseph Mugaanyi, Shengdong Wu, Caide Lu

**Affiliations:** Department of Hepatopancreatobiliary Surgery, Ningbo Medical Centre Lihuili Hospital, Ningbo University, Ningbo, 315040 Zhejiang China

**Keywords:** Cancer models, Hepatocellular carcinoma

## Abstract

The presence of microvascular invasion (MVI) is a critical determinant of early hepatocellular carcinoma (HCC) recurrence and prognosis. We developed a nomogram model integrating clinical laboratory examinations and radiological imaging results from our clinical database to predict microvascular invasion presence at preoperation in HCC patients. 242 patients with pathologically confirmed HCC at the Ningbo Medical Centre Lihuili Hospital from September 2015 to January 2021 were included in this study. Baseline clinical laboratory examinations and radiological imaging results were collected from our clinical database. LASSO regression analysis model was used to construct data dimensionality reduction and elements selection. Multivariate logistic regression analysis was performed to identify the independent risk factors associated with MVI and finally a nomogram for predicting MVI presence of HCC was established. Nomogram performance was assessed via internal validation and calibration curve statistics. Decision curve analysis (DCA) was conducted to determine the clinical usefulness of the nomogram model by quantifying the net benefits along with the increase in threshold probabilities. Survival analysis indicated that the probability of overall survival (OS) and recurrence-free survival (RFS) were significantly different between patients with MVI and without MVI (*P* < 0.05). Histopathologically identified MVI was found in 117 of 242 patients (48.3%). The preoperative factors associated with MVI were large tumor diameter (*OR* = 1.271, *95%CI*: 1.137–1.420, *P* < 0.001), AFP level greater than 20 ng/mL (20–400 vs. ≤ 20, *OR* = 2.025, *95%CI*: 1.056–3.885, *P* = 0.034; > 400 vs. ≤ 20, *OR* = 3.281, *95%CI*: 1.661–6.480, *P* = 0.001), total bilirubin level greater than 23 umol/l (*OR* = 2.247, *95%CI*: 1.037–4.868, *P* = 0.040). Incorporating tumor diameter, AFP and TB, the nomogram achieved a better concordance index of 0.725 (95%CI: 0.661–0.788) in predicting MVI presence. Nomogram analysis showed that the total factor score ranged from 0 to 160, and the corresponding risk rate ranged from 0.20 to 0.90. The DCA showed that if the threshold probability was > 5%, using the nomogram to diagnose MVI could acquire much more benefit. And the net benefit of the nomogram model was higher than single variable within 0.3–0.8 of threshold probability. In summary, the presence of MVI is an independent prognostic risk factor for RFS. The nomogram detailed here can preoperatively predict MVI presence in HCC patients. Using the nomogram model may constitute a usefully clinical tool to guide a rational and personalized subsequent therapeutic choice.

## Introduction

Hepatocellular carcinoma (HCC) is the sixth (4.7%) most commonly diagnosed cancer and the third (8.3%) leading cause of cancer-related death in the world according to global cancer statistics 2020^1^. Although HCC can be treated by surgical resection, ablation and liver transplantation in the early stage, but the 5-year recurrence rate is as high as 70%, the long-term prognosis is still not ideal^2^. In 2015, age-standardized 5-year relative survival of HCC was only 12.1% in China^3^, and only a small proportion of patients with HCC are suited for curative surgery. For operable HCC, recurrence and metastasis were the main drivers of poor prognosis^4^.

Microvascular invasion (MVI) is defined as the presence of tumor cells within a vascular lumen lined by endothelium that is visible only by microscopy^5,6^, and considered a critical determinant of early recurrence and survival of HCC. Tumor cells can spread and metastasize in the liver to form a portal vein tumor thrombus or multiple lesions or distant metastasis with the presence of MVI^7^. Recurrence within 5 years developed in about 70% of HCC patients treated with curative surgical resection. Nevertheless, the 5-year recurrence rate of liver transplant was 10–20%, slightly better than surgical resection. Zhao et al.^8^ found that patients with MVI benefited from anatomical hepatectomy in terms of disease-free survival rate compared with non-anatomical hepatectomy. Upon further research, it was found that residual intrahepatic metastases were frequently considered as the main cause of early recurrences within 2 years of tumor resection. Furthermore, early recurrence depends on the aggressiveness of the primary tumor, particularly the likelihood for MVI and satellitosis^9,10^. Chan et al.^11^ developed a practical statistical method that allows clinicians to estimate the risk of early recurrence with pre and post-operative data included MVI. Multiple features of MVI carry prognostic significance for HCC. Some studies have indicated that high-risk MVI patients did significantly worse in regard to both recurrence and survival^12–14^.

At present, the diagnosis of MVI was determined on histopathological examination of the surgical specimens obtained after HCC resection or liver transplantation. Consequently, the effect of the histopathological diagnosis on preoperative decision making was limited^15^. An accurate preoperative estimation of MVI presence can help surgeons choose accurate surgical approaches and improve HCC patients’ prognosis based on risk–benefit assessment. However, clinical surgeons were faced with a challenge of accurately predicting MVI and finding a uniform scheme or standard for doing so^16^. A nomogram has been considered as a reliable tool to integrate and quantify significant risk factors for disease prognosis^17,18^. Therefore, the aim of our research was to construct a novel nomogram to predict the probability of occurrence of MVI in HCC patients.

## Results

### Patients characteristics and prognostic outcomes

**Figure 1:**
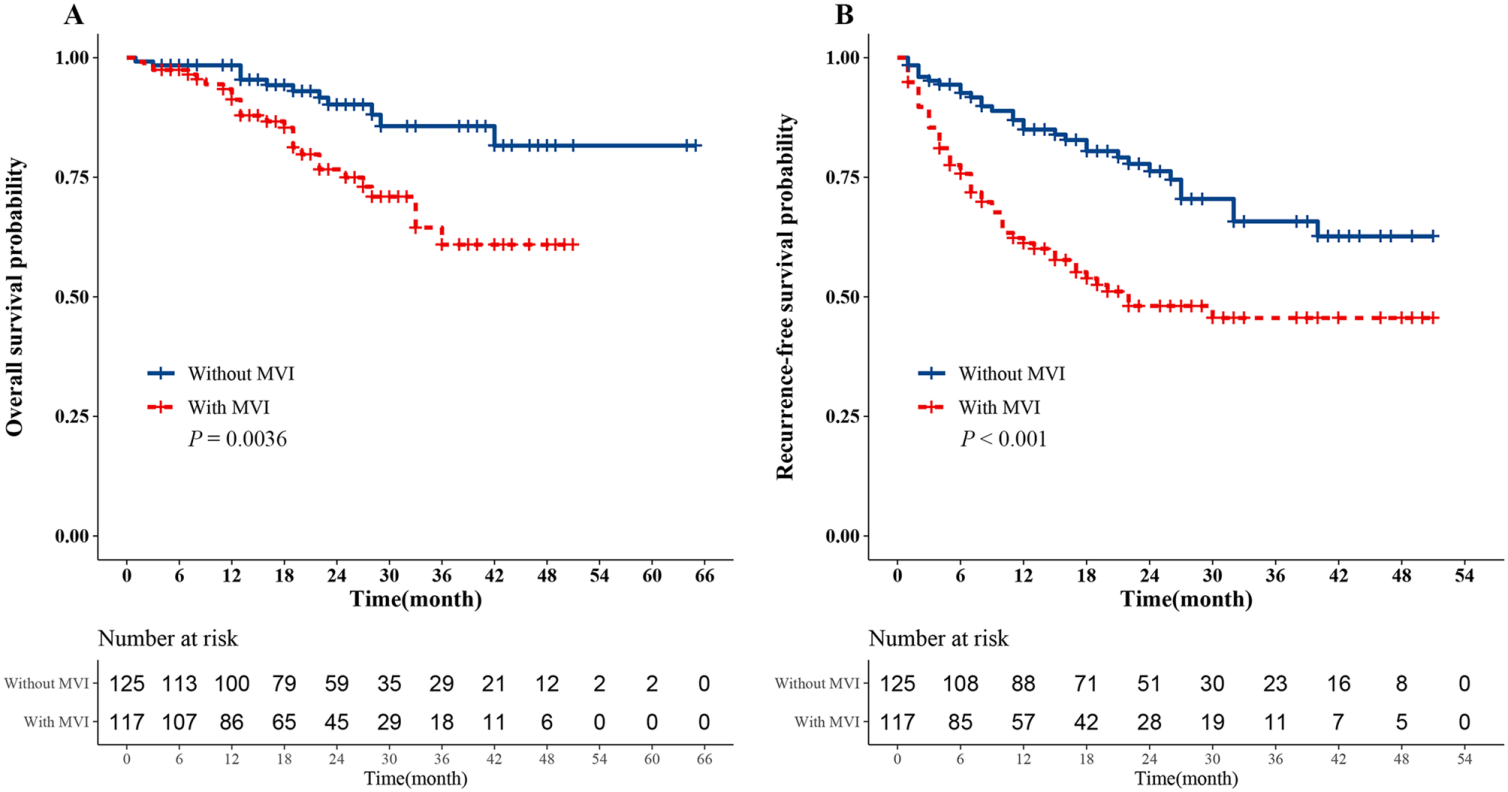
Kaplan–Meier survival curves analysis of histologic MVI in HCC. (**A**) Overall survival analysis (*P* = 0.0036). (**B**) Recurrence-free survival analysis (*P* < 0.001).

Survival analysis indicated that the probability of OS and RFS were significantly different between patients with MVI and without MVI (*P* < 0.05, Fig. 1). In patients with MVI, the 1-year and 3-year probability of OS were 91.2%, 60.9% respectively and 98.4%, 85.7%, respectively for without MVI. The 1-year and 3-year probability of RFS were 61.2%, 45.6%, respectively for patients with MVI and 85.0%, 65.8%, respectively for patients without MVI.

Univariate Cox regression showed that AST (> 50U/L), GGT (> 60U/L), platelets (> 125 × 109/L), PT (> 13.1 s), DB (> 8umol/l), AFP (> 400 ng/ml), MVI presence and tumor diameter are prognostic factors for OS while, HBV infection, AST (> 50U/L), AFP (> 400 ng/ml vs. ≤ 20 ng/ml), MVI presence and tumor diameter were prognostic factors for RFS (Table 1). The multivariate Cox regression analysis, PT (*HR* = 2.246, *95%CI*: 2.246–1.118, *P* = 0.023), AFP (> 400 ng/ml vs. ≤ 20 ng/ml: *HR* = 4.091, *95%CI*: 1.715–9.760, *P* = 0.001) and tumor diameter (*HR* = 1.123, *95%CI*: 1.038–1.216, *P* = 0.004) were determined to be the independent prognostic factors for OS, HBV infection (*HR* = 1.858, *95%CI*: 1.000–3.458, *P* = 0.05), AFP (> 400 ng/ml vs. ≤ 20 ng/ml: *HR* = 2.199, *95%CI*: 1.265–3.820, *P* = 0.005), MVI presence (*HR* = 1.780, *95%CI*: 1.085–2.920, *P* = 0.022) and tumor diameter (*HR* = 1.072, *95%CI*: 1.010–1.138, *P* = 0.023) for RFS (Table 2).

**Table 1:** Univariate Cox regression analysis for overall survival and tumor recurrence-free survival of HCC patients.

Variables	Overall survival			Tumor recurrence-free survival		
	*P* value	*HR*	*95%CI*	*P* value	*HR*	*95%CI*
Age	0.531	0.990	0.96–1.021	0.389	0.991	0.97–1.012
Gender	0.765	0.876	0.366–2.095	0.291	0.719	0.39–1.326
HBeAg	0.215	1.739	0.726–4.168	0.049	1.849	1.0003–3.418
**HBV DNA (IU/mL)**	0.472	1.268	0.664–2.42	0.678	0.909	0.581–1.423
≤ 10^3^ vs. Non-HBV	0.090	0.444	0.174–1.136	0.071	1.805	0.95–3.431
10^3^–10^5^ vs. Non-HBV	0.188	0.627	0.313–1.256	0.060	1.922	0.973–3.798
Family history of HCC	0.783	0.756	0.104–5.521	0.442	0.577	0.142–2.346
Anti-HBV therapy			–			–
Hypertension	0.383	0.706	0.323–1.543	0.819	0.943	0.57–1.56
Diabetes	0.626	0.773	0.274–2.18	0.690	1.133	0.614–2.089
Hypertension with diabetes	0.802	0.834	0.201–3.464	0.288	1.522	0.702–3.303
ALT (U/L)	0.990	0.996	0.494–2.008	0.517	0.849	0.517–1.393
AST (U/L)	0.035	1.993	1.051–3.778	0.003	1.945	1.263–2.994
GGT (U/L)	0.015	2.309	1.18–4.519	0.093	1.449	0.94–2.234
Platelets (× 10^9^/L)	0.047	0.523	0.276–0.991	0.341	0.801	0.508–1.263
TT (seconds)	0.987	1.006	0.488–2.072	0.767	0.927	0.56–1.533
PT (seconds)	0.042	2.034	1.025–4.035	0.970	0.990	0.573–1.709
TB (umol/l)	0.066	2.027	0.955–4.302	0.735	1.111	0.602–2.051
DB (umol/l)	0.020	2.216	1.134–4.333	0.203	1.395	0.836–2.328
**AFP (ng/mL)**						
20–400 vs. ≤ 20	0.075	2.404	0.915–6.318	0.054	1.768	0.991–3.156
> 400 vs. ≤ 20	< 0.001	4.607	1.958–10.841	< 0.001	3.042	1.807–5.120
**Imaging results**			–			
Tumor diameter (cm)	0.004	1.168	1.05–1.299	< 0.001	1.159	1.079–1.246
No. of tumors	0.845	0.928	0.439–1.961	0.036	1.630	1.033–2.571
Cirrhosis	0.753	1.112	0.575–2.15	0.600	0.885	0.561–1.396
**Pathologic diagnosis**						
MVI presence vs. absence	0.005	2.647	1.334–5.253	< 0.001	2.414	1.54–3.784
Tumor diameter (cm)	< 0.001	1.151	1.064–1.246	< 0.001	1.124	1.062–1.191
No. of tumors	0.350	1.397	0.693–2.818	0.063	1.568	0.975–2.522
Cirrhosis	0.514	2.047	0.239–17.571	0.763	0.835	0.257–2.706
T stage	< 0.001	2.010	1.508–2.681	< 0.001	1.651	1.361–2.002

*HbeAg* hepatitis B e antigen, *HBV* hepatitis B virus, *ALT* alanine aminotransferase, *AST* aspartate aminotransferase, *GGT* γ-glutamyl transpeptidase, *TT* thrombin time, *PT* prothrombin time, *TB* total bilirubin, *DB* direct bilirubin, *AFP* α-fetoprotein.

**Table 2:** Multivariate Cox regression analysis for overall survival and tumor recurrence-free survival of HCC patients.

Variables	Overall survival			Tumor recurrence-free survival		
	*P* value	*HR*	*95%CI*	*P* value	*HR*	*95%CI*
HBeAg	**–**	**–**	**–**	0.05	1.858	1.000–3.458
PT (seconds)	0.023	2.246	1.118–4.513	**–**	**–**	**–**
**AFP (ng/mL)**						
20–400 vs. ≤ 20	0.108	2.213	0.840–5.831	0.233	1.438	0.792–2.613
> 400 vs. ≤ 20	0.001	4.091	1.715–9.760	0.005	2.199	1.265–3.822
**Pathologic diagnosis**						
MVI presence vs. absence	**–**	**–**	**–**	0.022	1.780	1.085–2.920
Tumor diameter (cm)	0.004	1.123	1.038–1.216	0.023	1.072	1.010–1.138

*HbeAg* hepatitis B e antigen, *PT* prothrombin time, *AFP* α-fetoprotein.

The baseline characteristics of patients included are presented in Table 3. Histopathologically identifiable MVI was found in 117 of the 242 patients (48.3%). Univariate analysis showed that AST (> 40U/L vs. ≤ 40 U/L), GGT (> 60U/L vs. ≤ 60U/L), AFP and tumor diameter were significantly different between the two groups (*P* < 0.05, Table 3).

**Table 3:** Univariate analysis of MVI presence based on preoperative data in HCC patients.

Variables	Without MVI (n = 125)	MVI (n = 117)	*T/χ*^*2*^	*P* value
Age	60.4 ± 10.5	59.7 ± 10.4	0.13	0.719
**Gender**				
Male	106 (84.8)	91 (77.8)	1.97	0.161
Female	19 (15.2)	26 (22.2)		
**HBeAg**				
Positive	103 (82.4)	89 (76.1)	1.48	0.224
Negative	22 (17.6)	28 (23.9)		
**HBV DNA (IU/mL)**				
Non-HBV	22 (17.6)	28 (23.9)	2.55	0.279
≤ 10^3^	74 (56.8)	55 (47.0)		
10^3^–10^5^	32 (25.6)	34 (29.1)		
**Anti-HBV therapy**				
No	66 (52.8)	71 (60.7)		
Yes	59 (47.2)	46 (39.3)		
**Family history of HCC**				
No	119 (95.2)	112 (95.7)	0.04	0.844
Yes	5 (4.8)	5 (4.3)		
**Hypertension**				
No	98 (78.4)	82 (70.1)	2.19	0.139
Yes	27 (21.6)	35 (29.9)		
**Diabetes**				
No	109 (87.2)	101 (86.3)	0.04	0.841
Yes	16 (12.8)	16 (13.7)		
**Hypertension with diabetes**				
No	117 (93.6)	110 (94.0)	0.02	0.893
Yes	8 (6.4)	7 (6.0)		
**ALT (U/L)**				
≤ 50	94 (75.2)	84 (71.8)	0.36	0.548
> 50	31 (24.8)	33 (28.2)		
**AST (U/L)**				
≤ 40	86 (68.8)	62 (53.0)	6.36	0.012
> 40	39 (31.2)	55 (47.0)		
**GGT (U/L)**				
≤ 60	76 (60.8)	53 (45.3)	5.83	0.016
> 60	49 (39.2)	64 (54.7)		
**Platelets (× 10**^**9**^**/L)**				
≤ 125	35 (28.0)	37 (31.6)	0.38	0.538
> 125	90 (72.0)	80 (68.4)		
**TT (seconds)**				
≤ 16.6 s	89 (71.2)	92 (78.6)	1.77	0.183
> 16.6 s	36 (28.8)	25 (21.4)		
**PT (seconds)**				
≤ 13.1 s	100 (80.0)	93 (79.5)	0.01	0.921
> 13.1 s	25 (20.0)	24 (20.5)		
**TB (umol/l)**				
≤ 23	111 (88.8)	95 (81.2)	2.76	0.097
> 23	14 (11.2)	22 (18.8)		
**DB (umol/l)**				
≤ 8	106 (84.8)	92 (78.6)	1.54	0.214
> 8	19 (15.2)	25 (21.4)		
**AFP (ng/mL)**				
≤ 20	69 (55.2)	38 (32.5)	16.08	< 0.001
20–400	34 (27.2)	35 (29.9)		
> 400	22 (17.6)	44 (37.6)		
**Imaging results**				
Tumor diameter (cm)	4.12 ± 2.24	5.75 ± 2.91	12.42	0.001
**No. of tumors**				
Solitary	94 (75.2)	88 (75.2)	< 0.001	0.998
Multiple	31 (24.8)	29 (24.8)		
**Cirrhosis**				
No	79 (63.2)	75 (64.1)	0.02	0.884
Yes	46 (36.8)	42 (35.9)		

*MVI* microvascular invasion, *HbeAg* hepatitis B e antigen, *HBV* hepatitis B virus, *ALT* alanine aminotransferase, *AST* aspartate aminotransferase, *GGT* γ-glutamyl transpeptidase, *TT* thrombin time, *PT* prothrombin time, *TB* total bilirubin; *DB* direct bilirubin, *AFP* α-fetoprotein.

### Dimensionality reduction and element selection

**Figure 2:**
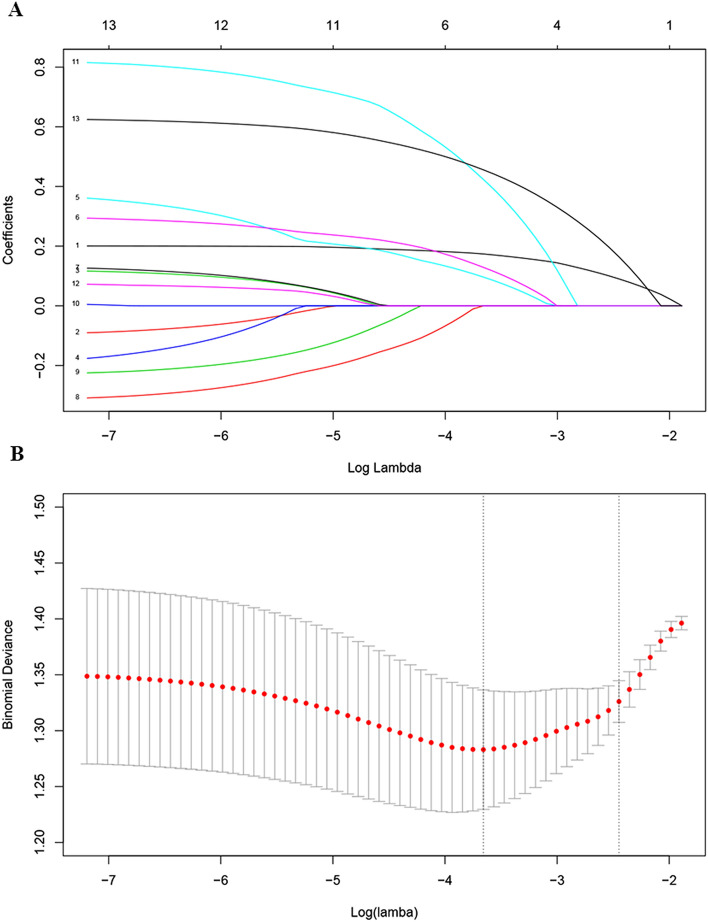
Nomogram model elements selection using the LASSO binary logistic regression model. (**A**) The LASSO coefficient profiles of the 13 features. AST, GGT, TB, AFP and tumor diameter were selected using LASSO binary logistic regression analysis. The LASSO coefficient profiles of the features were plotted. (**B**) The optimum parameter (lambda) selection in the LASSO model performed ten-fold cross-validation through minimum criteria. The partial likelihood deviance (binomial deviance) curve was presented versus log (lambda). Dotted vertical lines were showed at the optimum values by performing the lambda.min and the lambda.1se.

AST, GGT, TB, AFP and tumor diameter were selected using LASSO binary logistic regression analysis. The LASSO coefficient profiles of the features were plotted (Fig. 2A). The optimum parameter (lambda) selection in the LASSO model performed tenfold cross-validation through minimum criteria. The partial likelihood deviance (binomial deviance) curve was presented versus log (lambda). Dotted vertical lines were showed at the optimum values by performing the lambda.min and the lambda.1se (Fig. 2B). Finally, we chose the optimum value corresponding to the minimum value of lambda.

### Development and validation of the MVI-predicting nomogram

**Figure 3:**
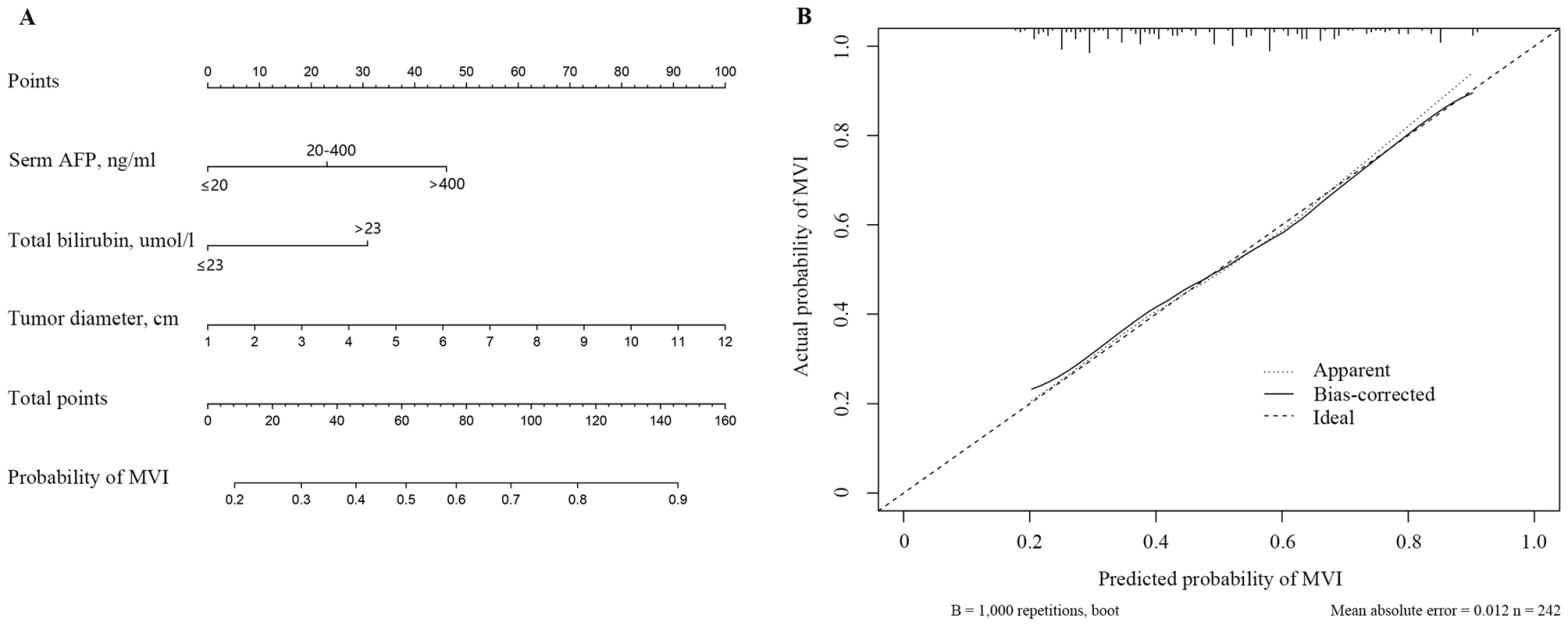
Developed diagnosis nomogram for microvascular invasion prediction. (**A**) A vertical line was drown upward to find the number of points received for AFP, tumor diameter and TB. The sum of three influencing factors was presented on the total point axis, and a vertical line was also drawn downward to the the probability of MVI. (**B**) The calibration curves of nomogram model prediction in HCC patients. The X-axis showed the predicted probability of MVI. The Y-axis showed the actual probability of MVI. The solid line indicated the performance of the developed nomogram model.

We incorporated the AST, GGT, TB, AFP and tumor diameter into the multivariate logistic regression equation. Multivariate logistic regression analysis indicated that the preoperative factors associated with MVI were large tumor diameter (*OR* = 1.271, *95%CI*: 1.137–1.420, *P* < 0.001), AFP level greater than 20 ng/mL (20–400 vs. ≤ 20, *OR* = 2.025, *95%CI*: 1.056–3.885, *P* = 0.034; > 400 vs. ≤ 20, *OR* = 3.281, *95%CI*: 1.661–6.480, *P* = 0.001), total bilirubin level greater than 23 umol/l (*OR* = 2.247, *95%CI*: 1.037–4.868, *P* = 0.040)(Table 4). Incorporating tumor diameter, AFP and TB, the nomogram (Fig. 3A) achieved a better concordance index of 0.725 (95%CI: 0.661–0.788) with 1000 bootstrap samples to measure discrimination in predicting MVI presence (Fig. 3B). The sensitivity and specificity were 76.8%, and 69.4%, respectively.

**Table 4:** Multivariate Logistic regression analysis of MVI presence based on preoperative data in HCC patients.

Variables	*β*	*P* value	*OR*	95%CI
**AFP (ng/mL)**				
20–400 vs. ≤ 20	0.706	0.034	2.025	1.056–3.885
> 400 vs. ≤ 20	1.188	0.001	3.281	1.661–6.480
**Imaging results**				
Tumor diameter (cm)	0.239	< 0.001	− 1.271	1.137–1.420
**TB**				
≤ 23 vs. > 23 (U/L)	0.810	0.040	2.247	1.037–4.868
**AST**				
≤ 40 vs. > 40 (U/L)	0.830	0.488	1.239	1.077–4.887
**GGT**				
≤ 60 vs. > 60 (U/L)	0.609	0.376	1.251	1.017–3.322

*AFP* α-fetoprotein, *TB* total bilirubin, *AST* aspartate aminotransferase, *GGT* γ-glutamyl transpeptidase.

**Figure 4:**
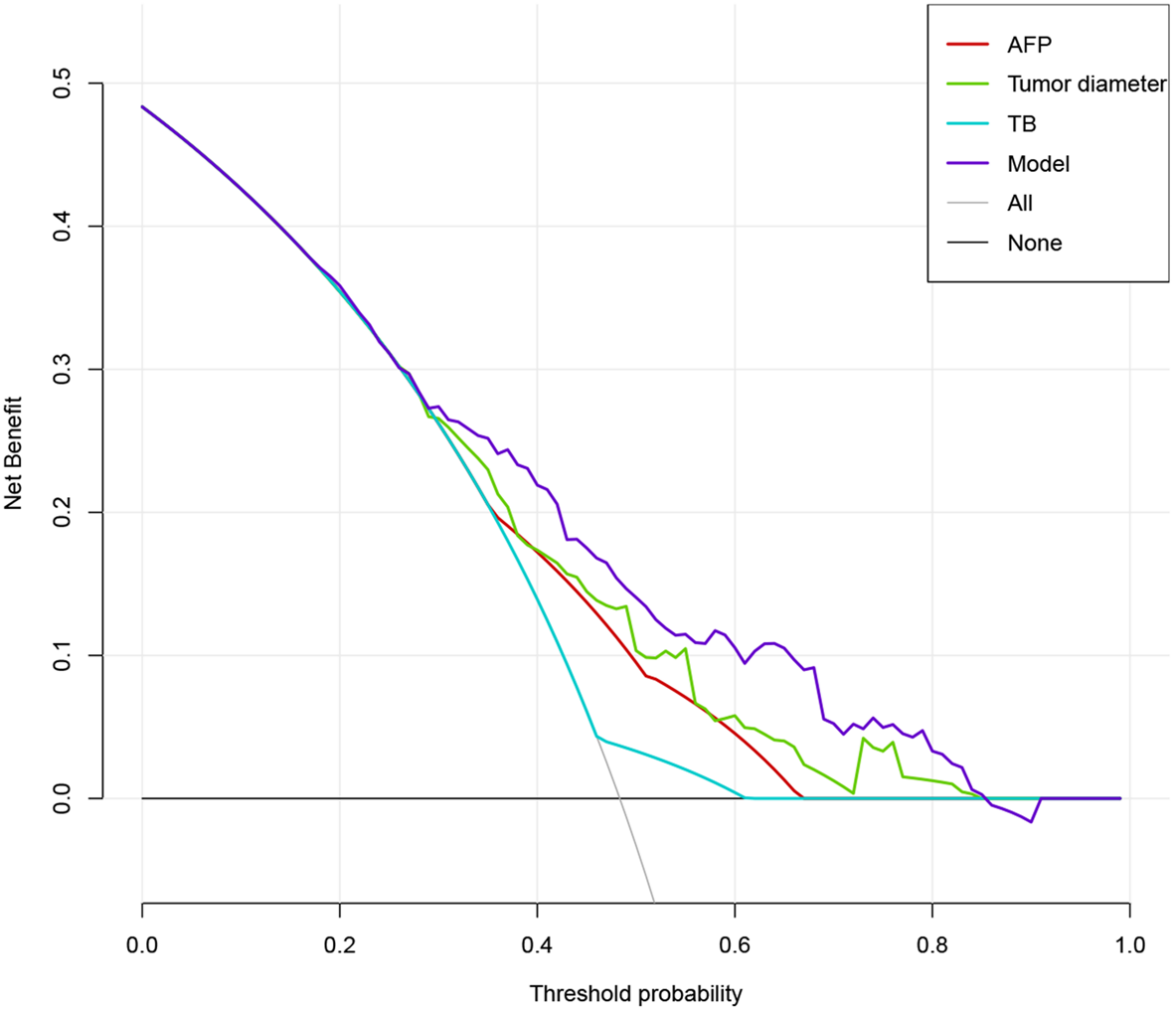
The decision curve analysis for developed nomogram model. The DCA demonstrated that if the threshold probability was > 5%, using the nomogram to diagnose MVI could acquired much more benefit. And the net benefit of the nomogram model was higher than single varirable within 0.3–0.8 of threshold probability.

DCA showed that if the threshold probability was > 5%, using the nomogram to diagnose MVI could be much more beneficial and it was obvious that its net benefit was considerably higher than that of independent clinical tumor diameter, AFP and TB models within 0.3–0.8 of threshold probability (Fig. 4).

## Discussion

According to statistical analysis of newly diagnosed HCC patients, patients with histopathologically identifiable MVI (48.3%) had a worse post hepatic resection prognosis and MVI was an independent risk factor for RFS. Incorporating three variables, tumor diameter, AFP and TB, screened through multivariate logistic regression, we built and validated a new preoperative prediction nomogram model for MVI in HCC patients. Individuals with higher total points were at greater risk for MVI. For example, if an HCC patient’s tumor diameter was 5 cm, AFP > 400 ng/ml, and TB > 23 umol/l, their total points were 116.5, and the corresponding MVI was about 80%; thus, the predicted probability of MVI in such a patient can be regarded as very high. The resultant nomogram could accurately preoperatively distinguish between patients with and without MVI and with better consistency between the predicted probability and the actual frequency of MVI. Anatomical resection or partial hepatic resection with a wide tumor margin was recommended to eradicate MVI^19^. And Hirokawa et al. found that the disease-free survival rate associated with surgical margin ≥ 10 mm was significantly better than that associated with surgical margin < 10 mm in MVI-positive patients^20^. Our nomogram provides an intuitive and easy-to-understand clinical tool to determine the risk of MVI for HCC patients.

Several studies have developed related preoperative prediction models for MVI in HCC. A preoperative prediction of MVI in HBV-related HCC within the Milan criteria indicated that the preoperative factors associated with MVI were large tumor diameter, multiple nodules, incomplete capsule, AFP level, platelet, HBV DNA load, and a typical dynamic pattern of tumors on contrast-enhanced MRI^15^. Pan et al.^7^ reported that tumor size, number of tumors, neutrophils and AFP were risk factors independently associated with MVI. A radiomic analysis of contrast-enhanced CT indicated that the nomogram model demonstrates good performance for predicting MVI and clinical outcomes with large-scale clinico radiologic and radiomic features^21^. Deng et al.^22^ found that when incorporating the independent risk factors of MVI including tumor size, AFP and neutrophil to lymphocyte ratio (NLR), the resulting nomogram achieved a concordance index of 0.71. Although several studies have developed and validated preoperative prediction models for MVI in HCC patients, the clinical features of the recruited patients in these studies were heterogeneous and so were the inclusion criteria. Consequently, further research was urgently needed to harmonize and improve the MVI prediction accuracy. In our study, tumor diameter and AFP were the independent MVI prognostic factors, consistent with previous studies. A study in a multicenter international database found that the incidence of MVI increased with tumor size of HCC resection patients (≤ 3 cm: 25%; 3.1–5 cm: 40%; 5.1–6.5 cm: 55%; > 6.5 cm: 63%)^23^. Vessels that encapsulate tumor clusters (VETC), previously linked to HCC metastatic dissemination, which was associated with high AFP levels and poor differentiation, and VETC was well correlated with MVI^24^. In addition, first we found that total bilirubin was also a significant predictive factor for MVI when integrated the tumor diameter and AFP. An elevated level of bilirubin almost always indicated the presence of an underlying abnormal liver function. The alteration of total bilirubin was significantly associated with the progression of liver cancer, and with the progression of HCC, most liver function indexes were gradually dysregulated^25^. According to a recent study, a preoperative radiomics-based nomogram demonstrated that the model provided better predictive performance when integrated AFP and TB. However, this was only found in patients with solitary hepatocellular carcinoma ≤ 5 cm^26^. In very early and early HCC patients, TB was also a significant risk factor for overall survival^27^ and disease-free survival prediction model^28^, but the mechanism of interaction between TB and MVI was still not very clear.

Athough preoperative clinical biochemical results and radiological imaging were usually selected in prediction model design, in our study, we choose the most frequently reported indicators, tumor diameter, number of tumors and cirrhosis from our clinical database for the potential advantages in standardization and popularization. Although 70–90% of HCC cases are associated with HBV infection in East Asia, especially in China^29,30^, chronic inflammation, suppression of local immune surveillance and high expression of metastasis-associated protein 1 (MTA1) played a significant role in MVI induced by HBV infection^4^. It is important to note that the rates of HBV infection and anti-HBV therapy had no difference between with MVI and without MVI patients in this study. We also found no correlation between MVI and HBV through multivariate analysis. Compared to other studies that limited the predicted population to HCC patients with HBV infection, our nomogram had a better application for new patients.

LASSO regression analysis model was used to construct data dimensionality reduction and element selection in our study. In previous studies, the LASSO regression had rarely been used in prediction models^7,15,21,22,31^. Overfitting, optimism, and miscalibration might be addressed and accounted for during the model development by applying bootstrapping techniques and LASSO regression^32^. Although the majority of previous studies generally randomly split the dataset into two subsets, a development sample and a validation sample, this was not done in our study. This approach was statistically inefficient or wasteful as not all available data was used in the development of the model^33^. Internal validation was a necessary part of prediction model development^34^. However, for external validation, substantial sample sizes should be used for sufficient power to detect clinically important changes in performance as compared with the internally validated estimate^35^. Owing to the sample size limitation, we were constrained to internal validation for the nomogram model. We also opted to conduct a DCA to determine the clinical utility of our nomogram.

To mitigate limitations faced by our study, future external validations are necessary since this was single center retrospective cohort. Secondly, our nomogram was developed based only on and limited to the clinical database; and other prognosis-related factors and biomarkers need to be identified and incorporated to further advance its accuracy. The last but not the least, the use of the nomogram in predicting the risk of a patient harboring MVI is only a new methodology, because MVI status is not the only factor in deciding on therapeutic procedures for HCC patients; and the correlation between MVI and subsequent recurrence is far from definite.

## Methods

### Patients

242 newly diagnosed patients with pathologically confirmed HCC who underwent hepatic resection at Ningbo Medical Center Lihuili Hospital from September 2015 to January 2021 were included. The inclusion criteria were: (1) Pathologically diagnosed as HCC and MVI; (2) Child–Pugh A or B classification; (3) No evidence of extrahepatic metastasis; (4) Treated by intend to cure resection, which was defined as negative margins with no residual tumor based on the histological examination. The exclusion criteria were: (1) Received preoperative anti-cancer medication; (2) History of other cancers; (3) No pathologic diagnosis of MVI; (4) Presence of gross vascular invasion; (5) Incomplete clinical or follow-up data. The study was approved by the ethics committee of Ningbo Medical Centre Lihuili Hospital (Approval number: KY2021PJ060). All methods were performed in accordance with the relevant guideline and regulations. Informed consent was obtained from all HCC patients for their data to be used for the research.

### Preoperative examination and followed-up

Preoperative examination included laboratory biochemical and pathological examination, contrast-enhanced magnetic resonance imaging (CEMRI) and contrast-enhanced computed tomography (CECT). Laboratory biochemical included hepatitis B and C immunology, HBV DNA, alanine aminotransferase (ALT), aspartate aminotransferase (AST), γ-glutamyl transpeptidase (GGT), platelets, thrombin time (TT), prothrombin time (PT), total bilirubin (TB), direct bilirubin (DB) and α-fetoprotein (AFP). The cutoff value of biochemical biomarkers was set according to Health Industry Standard of the People's Republic of China published by National Health Commission of the People’s Republic of China. The cutoff value of AFP to 400 ng/mL was set by review previous HCC researches^15,23,26,36^. Patients were defined as hypertensive on the basis of ‘gold standard’, and had at least three consecutive measurements of systolic blood pressure (SBP) > 140 mm Hg and/or diastolic blood pressure (DBP) > 90 mm Hg 22. Controls had SBP and DBP < 120 mm Hg and < 80 mm Hg, and first degree relatives had no family history of hypertension. Type 2 diabetes mellitus (T2DM) was diagnosed according to the World Health Organization criteria: (1) Fasting glucose level > 7 mmol/l; or (2) the 2-h oral glucose tolerance test showing a glucose level of ≥ 11.1 mmol/l; or (3) hemoglobin A1c ≥ 6.5%, or (4) the subject has a clinical diagnosis of the disease. Anatomic or nonanatomic resection was performed after the clinical evaluation, and all the obtained surgical specimens were histologically assessed to determine the presence of MVI as well as the Edmondson-Steiner grade by different pathologists. Pathological examination results included tumor diameter, no. of tumors, cirrhosis and MVI. Radiological imaging (CECT and CEMRI) results included tumor diameter, no. of tumors and cirrhosis. Baseline data was collected from our hospital clinical database. Patients were consistently followed-up after HCC resection at intervals of 3 months. Patient follow-up was aimed at determination of overall survival (OS) and recurrence-free survival (RFS). OS was measured from the date of HCC resection to the date of the patient’s death or the date of last follow-up visit. RFS was calculated from the date of HCC resection to the date when tumor recurrence was diagnosed. The preoperative and tumor recurrence diagnosis were based on criteria of the guidelines for diagnosis and treatment of primary liver cancer in China^37^.

### Statistical analysis

Statistical analysis of the numerical variables was performed using unpaired Student’s t-test for parametric data, Categorical variables were compared using Pearson's chi-square test or Fisher exact test. Survival curves were calculated using the Kaplan–Meier method and performed using the log-rank test. Multivariate Cox proportional hazards regression model was used to evaluate the independent prognostic factors of overall survival and tumor recurrence. LASSO regression analysis model was used to construct data dimensionality reduction and element selection. Subsequently, stepwise multivariate logistic regression analysis was performed to identify the independent risk factors. Then, a nomogram was formulated to predict MVI based on the results of LASSO regression and multivariate logistic regression analysis. Nomogram performance was assessed via internal validation and calibration curve statistics (concordance index was calculated to measure discrimination with1000 bootstrapping techniques). Decision curve analysis (DCA) was conducted to determine the clinical usefulness of the nomogram by quantifying the net benefits along with the increase of threshold probabilities.

Student’s t-test, pearson's chi-square test or fisher exact test, survival analysis, and logistic regression analysis were performed using SPSS 25.0 (IBM Corporation, 2020, USA). LASSO regression, nomogram, survival figures and decision curve analysis were performed or plotted using R version 3.6.2 and all figures were plotted by R. (R: Language and Environment for Statistical Computing, R core Team, R foundation for Statistical Computing, Vinena, Austria, 2019, http://www.r-project.org/), with packages dependencies: “rms”, “glamet”, “rmda” “survival”, “survminer” and “pROC”. *P* < 0.05 was considered statistically significant.
