# Author Correction: Preoperative nomogram for microvascular invasion prediction based on clinical database in hepatocellular carcinoma

**DOI:** 10.1038/s41598-021-99553-w

**Published:** 2021-10-04

**Authors:** Shuqi Mao, Xi Yu, Yong Yang, Yuying Shan, Joseph Mugaanyi, Shengdong Wu, Caide Lu

**Affiliations:** Department of Hepatopancreatobiliary Surgery, Ningbo Medical Centre Lihuili Hospital, Ningbo University, Ningbo, 315040 Zhejiang China

Correction to: *Scientific Reports* 10.1038/s41598-021-93528-7, published online 07 July 2021

The original version of this Article contained an error in the Introduction,

"Hepatocellular carcinoma (HCC) is the seventh (4.7%) most commonly diagnosed cancer"

now reads:

"Hepatocellular carcinoma (HCC) is the sixth (4.7%) most commonly diagnosed cancer".

Additionally, the Article contained errors in the x-axis and y-axis of Figure 3B, where the x-axis,

“Nomogram Predicated Survival”

now reads:

“Predicted probability of MVI”

And, the y-axis,

“Actual Survival”

now reads:

“Actual probability of MVI”

Consequently, the legend of the Figure 3 has been corrected accordingly,

“Developed diagnosis nomogram for microvascular invasion prediction. (**A**) A vertical line was drown upward to find the number of points received for AFP, tumor diameter and TB. The sum of three influencing factors was presented on the total point axis, and a vertical line was also drawn downward to the the probability of MVI. (**B**) The calibration curves of nomogram model prediction in HCC patients. The X-axis showed the predicted survival. The Y-axis showed the actual survival. The solid line indicated the performance of the developed nomogram model.”

now reads:

“Developed diagnosis nomogram for microvascular invasion prediction. (**A**) A vertical line was drown upward to find the number of points received for AFP, tumor diameter and TB. The sum of three influencing factors was presented on the total point axis, and a vertical line was also drawn downward to the the probability of MVI. (**B**) The calibration curves of nomogram model prediction in HCC patients. The X-axis showed the predicted probability of MVI. The Y-axis showed the actual probability of MVI. The solid line indicated the performance of the developed nomogram model.”

The original Figure 3 and accompanying legend appear below.

**Figure 3:**
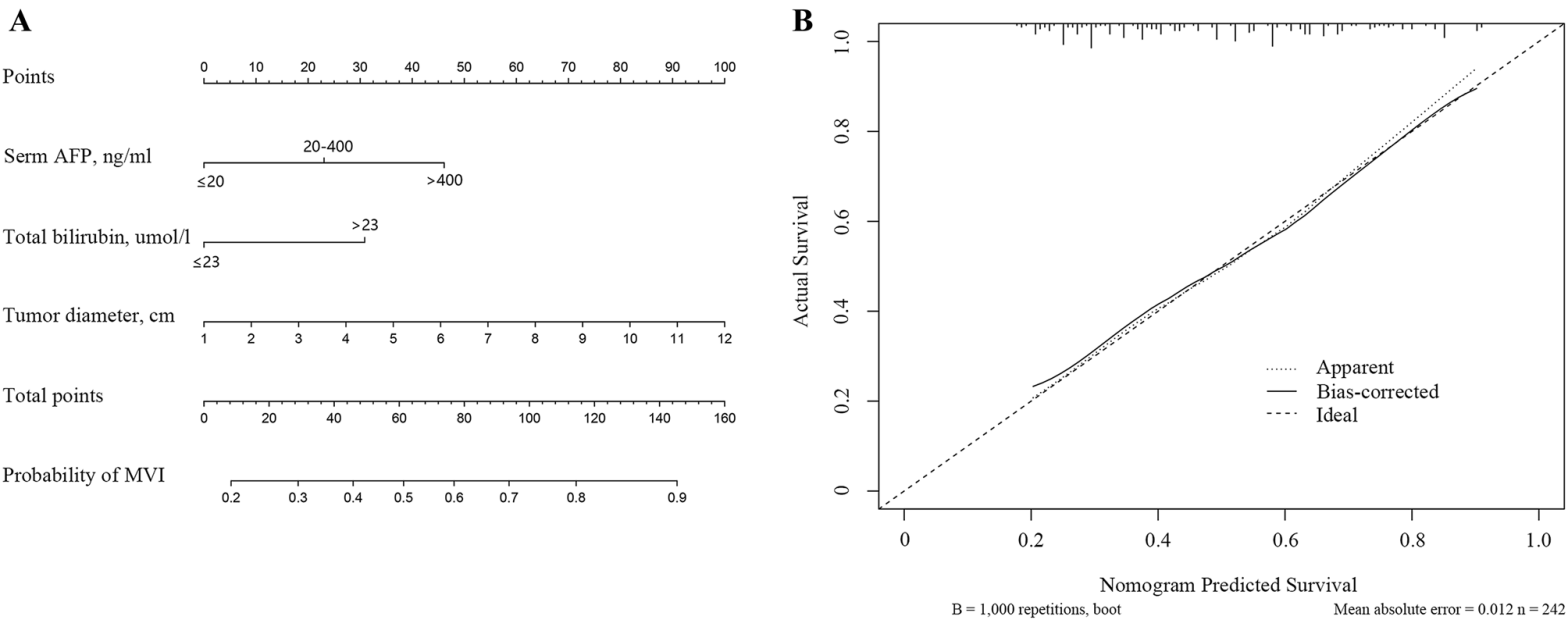
Developed diagnosis nomogram for microvascular invasion prediction. (**A**) A vertical line was drown upward to find the number of points received for AFP, tumor diameter and TB. The sum of three influencing factors was presented on the total point axis, and a vertical line was also drawn downward to the the probability of MVI. (**B**) The calibration curves of nomogram model prediction in HCC patients. The X-axis showed the predicted survival. The Y-axis showed the actual survival. The solid line indicated the performance of the developed nomogram model.

The original Article has been corrected.

